# Four new species of *Pseudoplagiostoma* (*Pseudoplagiostomataceae*, *Diaporthales*) based on morphology and multi-locus phylogeny from China

**DOI:** 10.3897/mycokeys.139.207860

**Published:** 2026-08-27

**Authors:** Jin-Yan Sheng, Yang Jiang, Xi-Gang Yan, Xing-Sheng Wang, Yue Zhang, Cong-Cong Ai, Xiu-Guo Zhang, Shi Wang

**Affiliations:** 1 College of Life Sciences, Shandong Normal University, Jinan, 250358, China College of Life Sciences, Shandong Normal University Jinan China; 2 Shandong Provincial Key Laboratory for Biology of Vegetable Diseases and Insect Pests, College of Plant Protection, Shandong Agricultural University, Taian, 271018, China Shandong Provincial Key Laboratory for Biology of Vegetable Diseases and Insect Pests, College of Plant Protection, Shandong Agricultural University Taian China

**Keywords:** *
Ascomycetes
*, new taxa, phylogenetic analysis, phytopathogenic fungi, taxonomy

## Abstract

*Pseudoplagiostoma* fungi are globally widespread, encompassing plant-pathogenic, saprophytic, and endophytic fungal species associated with diverse plant hosts. Four *Pseudoplagiostoma* isolates were recovered from diseased leaves of *Artocarpus hainanensis*, *Cinnamomum* sp., *Mallotus hookerianus*, and *Trachelospermum jasminoides* in China. These were identified and formally described in the present study. Species boundaries were delineated using an integrative approach combining molecular phylogenetic analyses and morphological comparisons with closely related taxa. Phylogenetic analyses were conducted on concatenated LSU, ITS, *RPB2*, *TEF1-α*, and *TUB2* sequence datasets using both maximum likelihood and Bayesian inference methods. The results demonstrated that these four isolates form well-supported, independent monophyletic lineages, clearly distinct from all previously described *Pseudoplagiostoma* species. Morphological characterization of colonies, conidiogenous cells, and conidia revealed stable phenotypic differences from closely related species, further corroborating their taxonomic novelty. Accordingly, four new species of *Pseudoplagiostoma* are proposed: *Ps. artocarpi*, *Ps. cinnamomi*, *Ps. malloti*, and *Ps. trachelospermi*.

## Introduction

The genus *Pseudoplagiostoma* Cheew., M.J. Wingf. & Crous was formally described by Cheewangkoon et al. (2010), with *Pseudoplagiostoma eucalypti* designated as the type species. The genus is placed in *Pseudoplagiostomataceae* (the family established concurrently with the genus; Hyde et al. 2024a), *Diaporthales*, *Sordariomycetes*, and *Ascomycota*, and the type species was originally isolated from diseased leaves of *Eucalyptus* in Australia. To date, 36 nomenclatural records of *Pseudoplagiostoma* are listed in Index Fungorum (http://www.indexfungorum.org/, accessed 1 June 2026). Morphologically, *Pseudoplagiostoma* resembles *Plagiostoma* and *Diaporthe* but differs markedly in the mode of conidiogenesis and conidial septation. Members of *Pseudoplagiostoma* exhibit distinct morphological features in both sexual and asexual morphs. The sexual morph is characterized by immersed, rostrate ascomata with an ostiole and unitunicate asci that bear a non-amyloid subapical ring. Its ascospores are hyaline, either aseptate or single-septate close to the median region, and furnished with elongated, hyaline terminal appendages. The asexual morph possesses both superficial and immersed conidiomata containing clusters of apically proliferating conidiogenous cells. The conidia produced are hyaline and ellipsoidal, whereas distinct conidiophores are absent in this morph (Cheewangkoon et al. 2010; Suwannarach et al. 2016; Mu et al. 2022; Zhang et al. 2023). With advances in molecular techniques, multi-locus phylogenetic analyses have provided robust evolutionary evidence to revise and refine traditional phenotype-based classification frameworks (Liu et al. 2016; Hyde et al. 2020). Species of *Pseudoplagiostoma* are not exclusively phytopathogenic and occupy diverse ecological niches: some live as endophytes within asymptomatic plant tissues, some serve as saprobes decomposing dead plant litter, and others act as phytopathogens causing leaf spots, twig diebacks, and fruit rots (Cao et al. 2023; Negi et al. 2024). This genus colonizes a broad range of host plants across numerous plant families, including *Myrtaceae*, *Lauraceae*, *Fagaceae*, *Bambusoideae*, *Dipterocarpaceae*, and *Anacardiaceae* (Crous et al. 2012a, 2012b; Bezerra et al. 2019; Tang et al. 2022; Zhang et al. 2023).

To date, approximately 32 valid *Pseudoplagiostoma* species have been described globally, with a continuous increase in the number of novel taxa reported from tropical and subtropical regions of Asia and South America in recent years (Silva et al. 2023; Jiang et al. 2025). Comprehensive field surveys throughout southern China were performed to explore the local fungal diversity and discover undocumented fungal resources. In this study, multiple *Pseudoplagiostoma* strains were isolated from symptomatic leaves of four host plants, *Artocarpus hainanensis*, *Cinnamomum* sp., *Mallotus hookerianus*, and *Trachelospermum jasminoides*, all collected within China. By integrating morphological observations and multi-locus phylogenetic inference, four new species of *Pseudoplagiostoma* are herein described and illustrated. The present findings broaden the documented species diversity of *Pseudoplagiostoma* and enrich the understanding of the taxonomic traits and ecological characteristics of this geographically widespread fungal genus.

## Materials and methods

### Sample collection and isolation

The isolates used in this study were obtained from diseased or rotted leaves collected in China from October to December 2025. Collection details were recorded (Rathnayaka et al. 2025), and the samples were taken to the laboratory at Shandong Normal University. Although a variety of fungi were present on the leaf samples, pure culture colonies were obtained by single-spore isolation and tissue isolation methods (Wang et al. 2023; Zhang et al. 2025). Single-spore isolation was conducted as described below, following the outline proposed by Senanayake et al. (2020). Conidia were evenly distributed over water agar and incubated at a constant temperature of 25 °C. Using a thin sterile needle under a stereomicroscope, single germinating conidia were collected and transferred to newly prepared potato dextrose agar (PDA). Leaf tissues measuring 5 mm × 5 mm were cut from diseased and rotted leaf lesions, and each tissue segment was placed in an individual sterile vessel. To achieve surface disinfection, leaf samples were soaked in 75% ethanol for 1 min prior to a single rinse with sterile water. Subsequently, tissues were exposed to 5% sodium hypochlorite solution for 30 s, and the samples were washed three times with sterile water. Once the surface water was absorbed by sterile filter paper, four leaf explants were placed symmetrically on PDA (200 g potato, 20 g dextrose, and 20 g agar dissolved in 1000 mL of distilled water; pH = 7.0), with the lesioned surface laid against the medium. All culture dishes were sealed and labeled before incubation at 25 °C. Fungal growth was monitored daily. Agar plugs carrying hyphae taken from the edge of fungal colonies were transferred to fresh PDA for strain purification, and subcultivation lasted 1–2 weeks.

### Morphological and cultural characterization

Colony morphologies were documented via photography on days 7 and 14 of incubation using a Canon Powershot G7X digital camera. Morphological examinations were carried out using sporulating fungal isolates cultivated on PDA. Sterile inoculation needles were used to collect conidial masses growing on PDA, which were subsequently transferred onto glass slides. A lactic acid-phenol mounting liquid (composed of lactic acid, phenol, glycerol, and sterile water mixed at a volume ratio of 1:1:2:1) was added to the samples, which were then covered with cover glasses to prepare temporary slides for microscopic observation. The temporary mounts were then examined under an Olympus BX53 light microscope equipped with an Olympus DP80 high-definition color digital camera to observe microscopic morphological features, including conidiophores, conidiogenous cells, and conidia. The Digimizer image analysis program (available at https://www.digimizer.com/) was used to quantify these microstructures, with a minimum of 15 replicates measured for each morphological feature. All fungal strains tested were preserved in 10% sterile glycerol solution at 4 °C to support subsequent experimental analyses. Dried cultures bearing conidial masses and corresponding fungal specimens generated in this study were deposited at the Herbarium Mycologicum Academiae Sinicae (**HMAS**). Living pure cultures were submitted to the Shandong Agricultural University Culture Collection (**SAUCC**). Taxonomic information on the new species was uploaded to MycoBank (http://www.mycobank.org/).

### DNA extraction, PCR amplification, and sequencing

Genomic DNA was extracted from actively growing fungal colonies cultured on PDA via the cetyltrimethylammonium bromide (CTAB) protocol described by Guo et al. (2000). DNA was amplified by polymerase chain reaction (PCR) using the following five loci: ITS, LSU, *RPB2*, *TEF1-α*, and *TUB2*. The primers used in the PCR and the reaction programs are shown in Table 1. Each 25 μL PCR reaction mixture consisted of 12.5 μL 2× Hieff Canace® Plus PCR Master Mix (supplied with dye; Yeasen Biotechnology, Shanghai, China, Cat. No. 10154ES03), 1 μL forward primer, 1 μL reverse primer, and 2 μL template genomic DNA. Sterile double-distilled water was added to a final volume of 25 μL. The purified PCR products were sequenced by Sangon Biotech Co., Ltd. (Shanghai, China). The sequencing data were analyzed and spliced using MEGA v. 7.0 (Kumar et al. 2016). Available sequence data representing known species of *Pseudoplagiostoma* were downloaded from GenBank (NCBI) following a review of the published literature. The retrieved reference sequences were combined with newly generated sequences from this study for phylogenetic analyses.

**Table 1.** Gene loci and corresponding PCR primers and programs used in this study.

**Locus**	**PCR primers**	**Sequence (5’ – 3’)**	**PCR cycles**	**References**
ITS	ITS5	GGA AGT AAA AGT CGT AAC AAG G	(94 °C: 30 s, 55 °C: 30 s, 72 °C: 45 s) × 29 cycles	White et al. 1990
	ITS4	TCC TCC GCT TAT TGA TAT GC		
LSU	LR0R	GTA CCC GCT GAA CTT AAG C	(94 °C: 30 s, 48 °C: 50 s, 72 °C: 1 min) × 35 cycles	Vilgalys and Hester 1990
	LR5	TCC TGA GGG AAA CTT CG		
*RPB2*	fRPB2-5F	GGG GWG AYC AGA AGA AGG C	(94 °C: 45 s, 60 °C: 45 s, 72 °C: 2 min) × 55 cycles	Liu et al. 1999; Sung et al. 2007
	fRPB2-7R	CCC ATR GCT TGY TTR CCC AT	(94 °C: 45 s, 54 °C: 45 s, 72 °C: 2 min) × 30 cycles	
*TEF1α*	EF1-728F	CAT CGA GAA GTT CGA GAA GG	(95 °C: 30 s, 48 °C: 30 s, 72 °C: 1 min) × 35 cycles	O’Donnell et al. 1998; Carbone and Kohn 1999
	EF-2	GGA RGT ACC AGT SAT CAT GTT		
*TUB2*	Bt-2a	GGT AAC CAA ATC GGT GCT GCT TTC	(95 °C: 30 s, 56 °C: 30 s, 72 °C: 1 min) × 35 cycles	Glass and Donaldson 1995
	Bt-2b	ACC CTC AGT GTA GTG ACC CTT GGC		

### Phylogenetic analyses

In this study, all novel sequences generated in this work were deposited in the NCBI GenBank database. Reference sequences for comparative analysis were retrieved from the National Center for Biotechnology Information (NCBI), following the latest taxonomic literature focused on the genus *Pseudoplagiostoma*. The newly acquired sequences were aligned with homologous sequences downloaded from GenBank using the web-based MAFFT 7 program under the Auto alignment strategy (Katoh et al. 2019), and the raw alignment matrix was manually trimmed and refined in BioEdit (Hall 2006). Ingroup taxa consisted of all fungal isolates obtained in the present study. Following the phylogenetic framework proposed by Jiang et al. (2025), species belonging to the closely related genus *Pyrispora* were selected as outgroups. Bayesian inference (BI) and maximum likelihood (ML) algorithms were both implemented to infer phylogenetic topologies based on the multi-locus concatenated matrix. Optimal nucleotide substitution models for each gene partition were selected in MrModeltest v. 2.3 under the Akaike information criterion (AIC), with the corresponding parameters set before BI. ML and BI analyses were run online via the CIPRES Science Gateway (https://www.phylo.org/, accessed 3 June 2026) or using local offline pipelines (Miller et al. 2012). ML trees were inferred in RAxML-HPC2 on XSEDE v. 8.2.12 with 1000 rapid bootstrap replicates under the GTR model and default settings (Stamatakis 2014). BI analyses were performed under the GTR+I+G model with sampling every 1000 generations, and runs ceased when the average standard deviation of split frequencies fell below 0.01 (Ronquist and Huelsenbeck 2003; Ronquist et al. 2012). Output phylogenetic trees were visualized using Interactive Tree Of Life (iTOL) v. 7.2 (https://itol.embl.de, accessed 8 June 2026). Phylogenetic trees were adjusted and refined using Adobe Illustrator CC 2024 (Letunic and Bork 2021). Comprehensive taxonomic information on all taxa included in this phylogenetic analysis is summarized in Suppl. material 1: table SS1.

## Results

### Phylogenetic analyses

**Figure 1.**
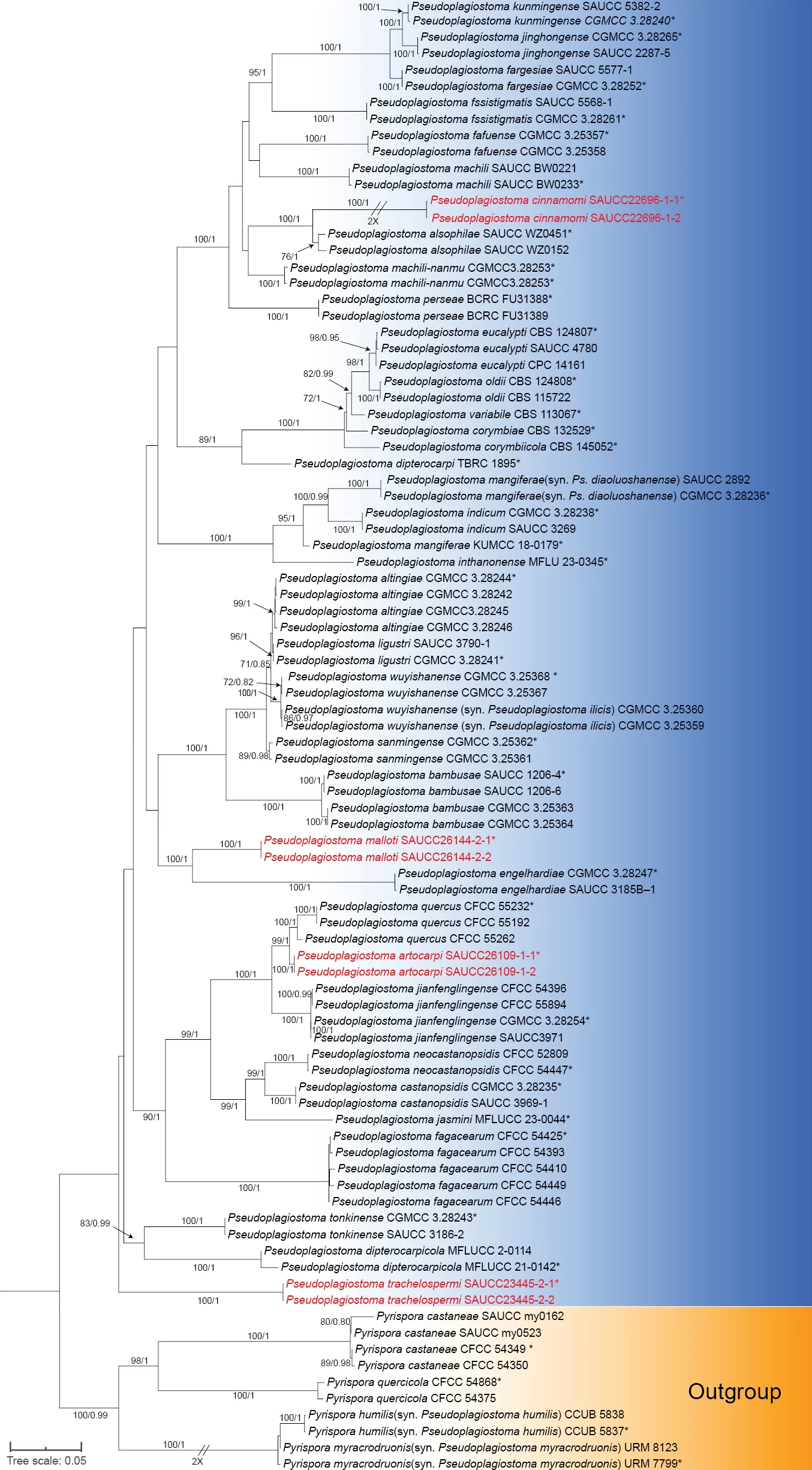
Maximum likelihood tree based on a combined dataset of ITS, LSU, *RPB2*, *TEF1-α*, and *TUB2* sequences. Maximum likelihood bootstrap support values (MLBS; left, ≥ 70%) and Bayesian posterior probabilities (BYPP; right, ≥ 0.80) are displayed above each node in the format MLBS/BYPP. Strains marked with an asterisk (*) correspond to ex-type or ex-epitype isolates. The *Pseudoplagiostoma* clade is shaded in blue, and the outgroup genus *Pyrispora* is highlighted within the yellow box. Strains newly isolated in the present study are printed in red font. The scale bar at the bottom represents 0.05 nucleotide substitutions per site. For a clearer layout of the phylogenetic tree, several long branches are truncated and labeled with two diagonal slashes (“//”) to indicate branch shortening.

Five molecular loci, ITS, LSU, *RPB2*, *TEF1-α*, and *TUB2*, were concatenated to clarify interspecific evolutionary relationships within the genus *Pseudoplagiostoma*. Following Jiang et al. (2025), strains of the closely related genus *Pyrispora* were selected as outgroups. The multi-locus alignment contained a total of 90 taxa, including four *Pyrispora* outgroup taxa: *Pyrispora castaneae*, *P. quercicola*, *P. humilis* (syn. *Pseudoplagiostoma humilis*), and *P. myracrodruonis*. A total of 4,123 characters, including gaps, were included in the phylogenetic analysis, viz. 1–690 for ITS, 691–1,565 for LSU, 1,566–2,364 for *TEF1-α*, 2,365–2,936 for *TUB2*, and 2,937–4,123 for *RPB2*. Of all characters, 2,420 were constant, 105 variable characters were parsimony-uninformative, and 1,598 characters were parsimony-informative. For Bayesian analyses, independent nucleotide substitution models were selected for each gene partition: GTR+I+G was assigned to ITS, *TEF1-α*, and *TUB2*, SYM+I+G to LSU, and GTR+G to *RPB2*. Phylogenetic topologies generated from maximum likelihood (ML) and Bayesian inference (BI) analyses were highly congruent, reliably reflecting the evolutionary framework of *Pseudoplagiostoma*. Branches in Fig. 1 are annotated with ML bootstrap support values (MLBS) ≥ 70% and Bayesian posterior probabilities (BYPP) ≥ 0.8. The 90 sampled isolates clustered into 40 distinct species-level lineages corresponding to previously published taxa of *Pseudoplagiostoma*. Phylogenetic analysis revealed that the eight isolates collected in this study formed four well-defined clades representing four novel species within *Pseudoplagiostoma*. Each of the four novel species clades recovered in this study received strong statistical support (MLBS ≥ 90%, BYPP = 1.0) (Fig. 1).

### Taxonomy

#### Pseudoplagiostoma artocarpi

Taxon classificationFungiDiaporthalesPseudoplagiostomataceae

J. Y. Sheng, X. G. Zhang & S. Wang sp. nov.

45598E64-A294-5C63-BEC6-FA54FA6A0401

864560

Fig. 2

##### Type.

China • Hainan Province: Ledong Li Autonomous County, on diseased leaves of *Artocarpus hainanensis* Merr. & Chun., 13 December 2025, J.Y. Sheng (HMAS 354639, holotype), ex-type living culture SAUCC26109-1-1 = SAUCC26109-1-2.

**Figure 2.**
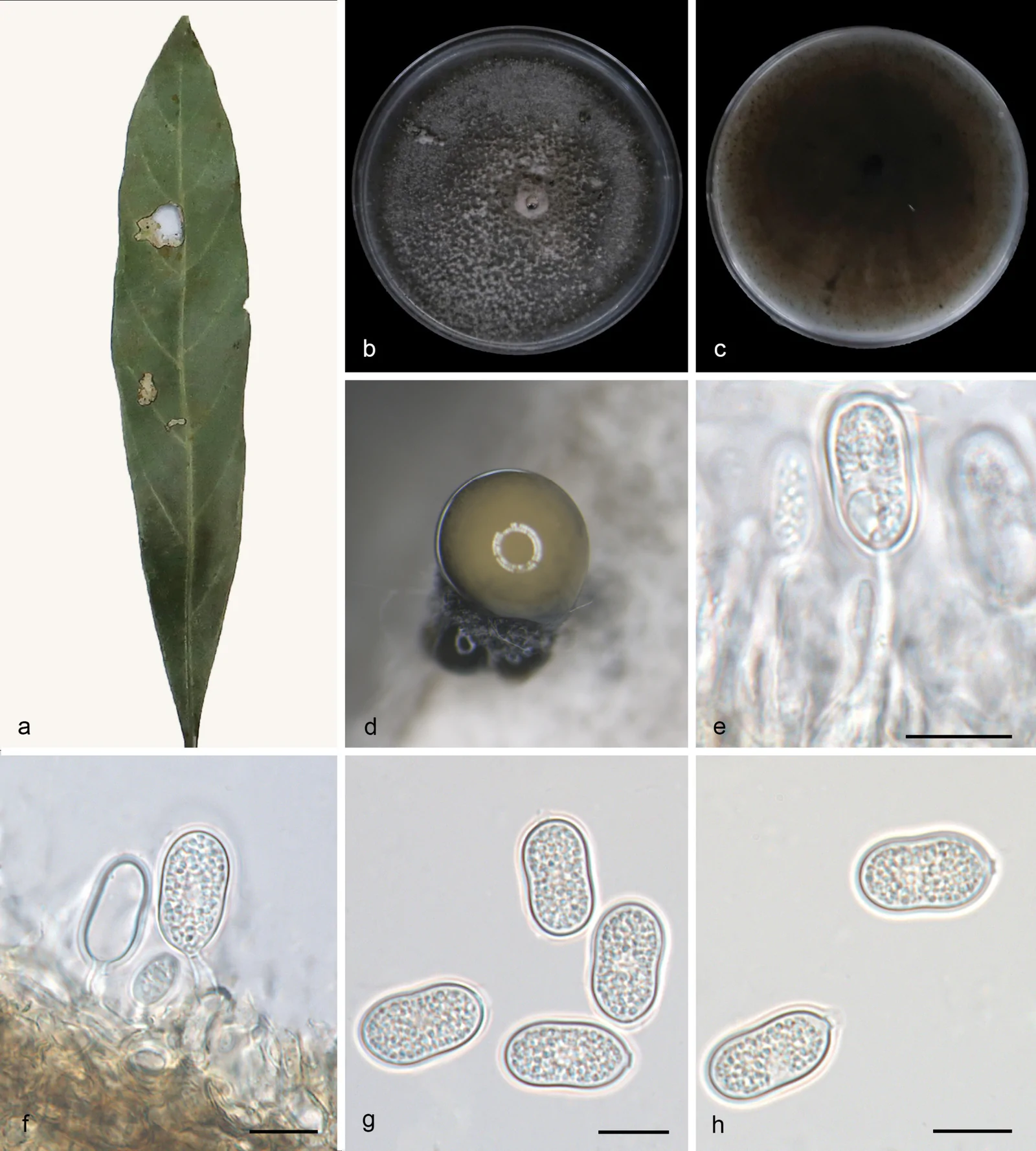
*Pseudoplagiostoma artocarpi* (SAUCC26109-1-1, ex-type). **a**. A diseased leaf of *Artocarpus hainanensis*; **b, c**. Colony on PDA from above and below after 14 days; **d**. Colony overview; **e, f**. Conidiogenous cells with conidia; **g, h**. Conidia. Scale bars: 10 μm (**e–h**).

##### Etymology.

Refers to the genus *Artocarpus*, from which the fungus was isolated.

##### Description.

Asexual morph: Conidiomata pycnidial, solitary, erumpent, globose, dark brown to black at base with pale yellowish-amber ostiolar disc. Conidiophores indistinct, often reduced to conidiogenous cells. Conidiogenous cells hyaline, smooth, lageniform to ampulliform, tapering toward the apex, phialidic, 6.2–13.8 × 2.1–3.0 μm (x̄ = 9.0 × 2.4 μm, *n* = 15). Conidia holoblastic, aseptate, hyaline, smooth, thin-walled, densely granular inside, ellipsoid to broadly oval, straight to slightly obovate, base tapering to a subtle flat scar, 12.4–20.0 × 7.3–9.8 μm (x̄ = 16.1 × 8.5 μm, *n* = 55). Sexual morph: Not observed.

##### Culture characteristics.

Colonies were incubated on PDA at 25 °C under dark conditions. After 14 days of incubation, the colonies attained a diameter of 74–82 mm, with a daily growth rate ranging from 5.3 to 5.9 mm. Colonies grew outward in an irregular pattern and were covered with abundant white aerial mycelium. As incubation continued, the colony center gradually turned yellowish-brown from white, and this pigmentation later spread to the periphery. The reverse side was white at first and finally became orange. Sparse sporulation was observed on PDA after 14 days of incubation.

##### Notes.

Phylogenetic analysis based on combined multi-locus sequences (ITS, LSU, *RPB2*, *TEF1-α*, and *TUB2*) revealed that *Pseudoplagiostoma artocarpi* (SAUCC26109-1-1 and SAUCC26109-1-2) clustered together and formed a distinct monophyletic subclade with full support (MLBS/BYPP = 100/1) within the genus *Pseudoplagiostoma*. This taxon exhibited a close phylogenetic affinity to *Ps. quercus* (CFCC 55232, CFCC 55192, and CFCC 55262). Distinct sequence divergences were detected between *Ps. artocarpi* and its closely allied species across all four loci (ITS, LSU, *TEF1-α*, and *TUB2*). *Pseudoplagiostoma artocarpi* (SAUCC26109-1-1, ex-type) differs from *Ps. quercus* (CFCC 55232, ex-type). Excluding gap sites, pairwise sequence comparisons revealed nucleotide differences of 1.06% (ITS, six out of 564 positions), 1.72% (LSU, 14 out of 814 positions), 2.47% (*TEF1-α*, 13 out of 527 positions), and 1.34% (*TUB2*, six out of 448 positions). Morphometric ranges partially overlap between *Ps. artocarpi* and its closely related species *Ps. quercus*. Even so, conidiogenous cells of *Ps. artocarpi* (SAUCC26109-1-1, living culture) tend to be shorter and narrower overall (6.2–13.8 × 2.1–3.0 μm) compared with those of *Ps. quercus* (CFCC 55232, living culture; 10–25 × 2–8.5 μm). Conidia of *Ps. artocarpi* (SAUCC26109-1-1) are generally smaller and thinner-walled (12.4–20.0 × 7.3–9.8 μm), whereas conidia of *Ps. quercus* measure (16–)17–21(–21.5) × (8.5–)9.5–11(–11.5) μm. Pycnidia of *Ps. artocarpi* bear a pale yellowish-amber ostiolar disc, while pycnidia of *Ps. quercus* are entirely black and lack pale ostiolar tissue. Conidia of *Ps. artocarpi* are densely granular internally without prominent guttules and possess inconspicuous flat basal scars. In contrast, *Ps. quercus* produces multi-guttulate conidia with a conspicuous hilum. Therefore, based on morphological distinctions and phylogenetic evidence, this fungus is proposed as *Pseudoplagiostoma artocarpi* sp. nov., following the species delimitation guidelines of Jeewon and Hyde (2016).

#### Pseudoplagiostoma cinnamomi

Taxon classificationFungiDiaporthalesPseudoplagiostomataceae

J. Y. Sheng, X. G. Zhang & S. Wang sp. nov.

A87B4D32-AB7B-5BBF-844D-3CAA84E55EFE

864562

Fig. 3

##### Type.

China • Fujian Province: Xinluo District, Longyan City, on diseased leaves of *Cinnamomum* sp., 18 October 2025, J.Y. Sheng (HMAS 354637, holotype), ex-type living culture SAUCC22696-1-1 = SAUCC22696-1-2.

**Figure 3.**
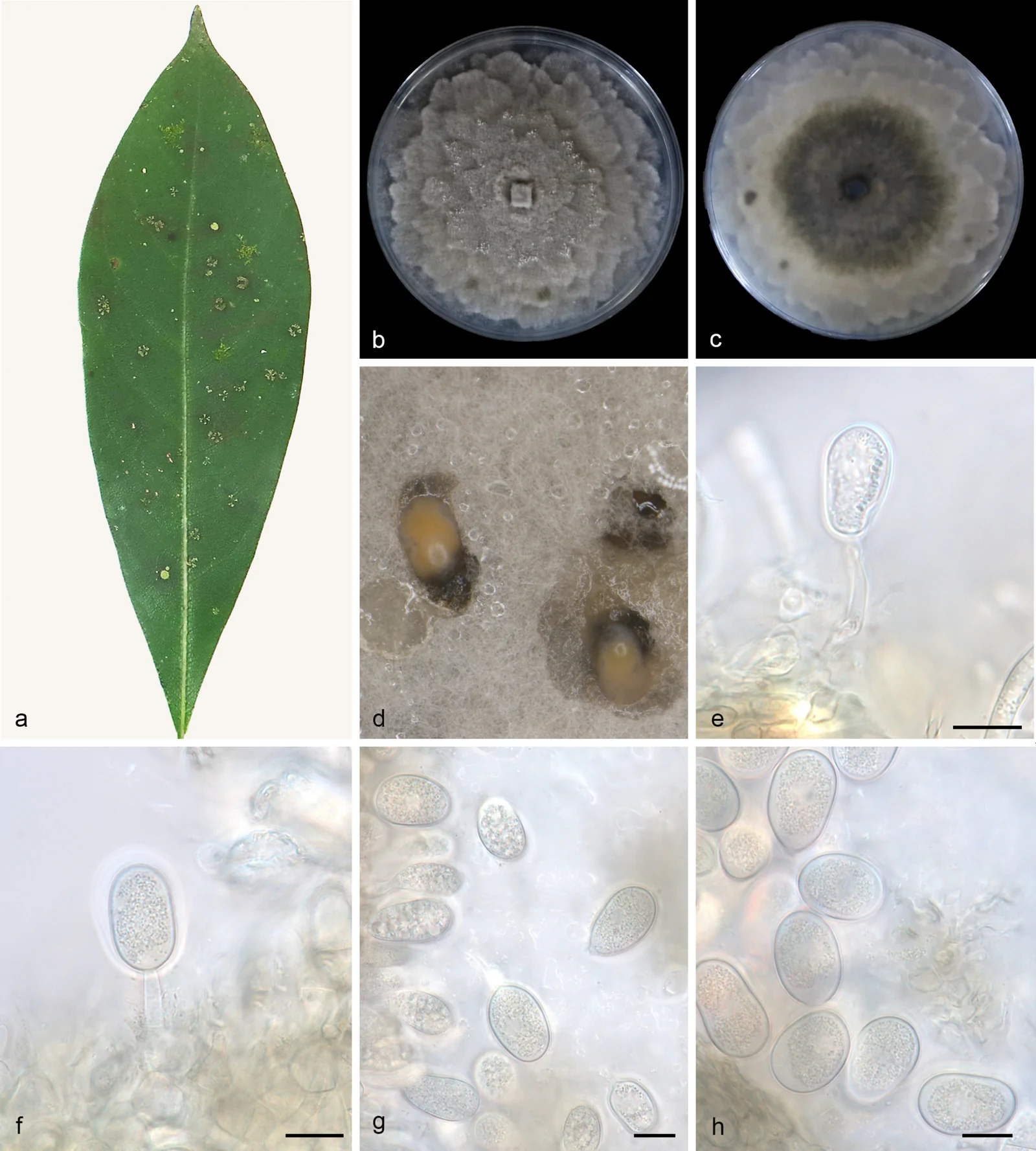
*Pseudoplagiostoma cinnamomi* (SAUCC22696-1-1, ex-type). **a**. A diseased leaf of *Cinnamomum* sp.; **b, c**. Colony on PDA from above and below after 14 days; **d**. Colony overview; **e, f**. Conidiogenous cells with conidia; **g, h**. Conidia. Scale bars: 10 μm (**e–h**).

##### Etymology.

Refers to the genus *Cinnamomum*, from which the fungus was isolated.

##### Description.

Asexual morph: Conidiomata pycnidial, solitary, erumpent, globose to pulvinate, pale brown to dark brown, exuding pale creamy conidial mass. Conidiophores indistinct, often reduced to conidiogenous cells. Conidiogenous cells hyaline, smooth, lageniform to ampulliform, tapering toward the apex, phialidic, 7.8–14.3 × 2.3–3.4 μm (x̄ = 11.1 × 3.1 μm, *n* = 15). Conidia holoblastic, aseptate, hyaline, smooth, thin-walled, densely granular, ellipsoid to broadly oval, straight to slightly obovate, base tapering to a subtle flat scar, 14.7–23.1 × 9.4–13.6 μm (x̄ = 19.1 × 12.1 μm, *n* = 50). Sexual morph: Not observed.

##### Culture characteristics.

Colonies were cultured on PDA medium at 25 °C in darkness. After 14 days of cultivation, colonies reached a diameter of 78–87 mm, with daily growth rates between 5.6 and 6.2 mm. Colonies grew outward in an irregular lobed pattern and were covered with dense fluffy white aerial mycelium. As incubation continued, the colony center gradually turned dark grayish-brown from white, and this pigmentation formed distinct concentric rings that spread outward toward the periphery. The reverse side was pale white at first and finally developed a prominent dark gray-brown central zone surrounded by off-white marginal areas. Sparse sporulation could be seen on PDA after 14 days of incubation, with small scattered pycnidia visible on the colony surface.

##### Notes.

Based on phylogenetic analysis combined with five loci (ITS, LSU, *RPB2*, *TEF1-α*, and *TUB2*), *Pseudoplagiostoma cinnamomi* (SAUCC22696-1-1 and SAUCC22696-1-2) constitutes an independent clade in the phylogenetic trees, closely related to *Ps. alstoniaeliae* (SAUCC WZ045 and SAUCC WZ20152). *Pseudoplagiostoma cinnamomi* (SAUCC22696-1-1, ex-type) is distinguished from *Ps. alstoniaeliae* (SAUCC WZ0451, ex-type) by nucleotide differences of 9/615 (1.46%), 1/808 (0.12%), 82/786 (10.43%), 23/507 (4.54%), and 8/470 (1.70%) characters in ITS, LSU, *RPB2*, *TEF1-α*, and *TUB2* sequences, respectively, with all gap sites excluded from the sequence comparisons. Morphologically, *Pseudoplagiostoma cinnamomi* produces only an asexual morph on host leaf tissue associated with leaf spots, and its sexual morph was not observed. The pycnidia of *Ps. cinnamomi* are pale brown to dark brown, contrasting with the entirely black pycnidia of *Ps. alstoniaeliae*. The conidiogenous cells of *Ps. cinnamomi* (7.8–14.3 × 2.3–3.4 μm) are generally wider than those of *Ps. alstoniaeliae* (8–13 × 1.5–3 μm). Conidia of *Ps. cinnamomi* are thin-walled and densely granular with subtle flat basal scars, whereas those of *Ps. alstoniaeliae* are guttulate and globose to irregularly ellipsoid and possess prominent peg-like hila at the base. Therefore, supported by morphological and phylogenetic data, this fungus is proposed as *Pseudoplagiostoma cinnamomi* sp. nov., following the species delimitation guidelines of Jeewon and Hyde (2016).

#### Pseudoplagiostoma malloti

Taxon classificationFungiDiaporthalesPseudoplagiostomataceae

J. Y. Sheng, X. G. Zhang & S. Wang sp. nov.

BC6782AD-35DF-579F-AFFF-5BDA3107BF66

864564

Fig. 4

##### Type.

China • Hainan Province: Ledong Li Autonomous County, on diseased leaves of *Mallotus hookerianus* Seem., 13 December 2025, J.Y.Sheng (HMAS 354640, holotype), ex-type living culture SAUCC26144-2-1 = SAUCC26144-2-2.

**Figure 4.**
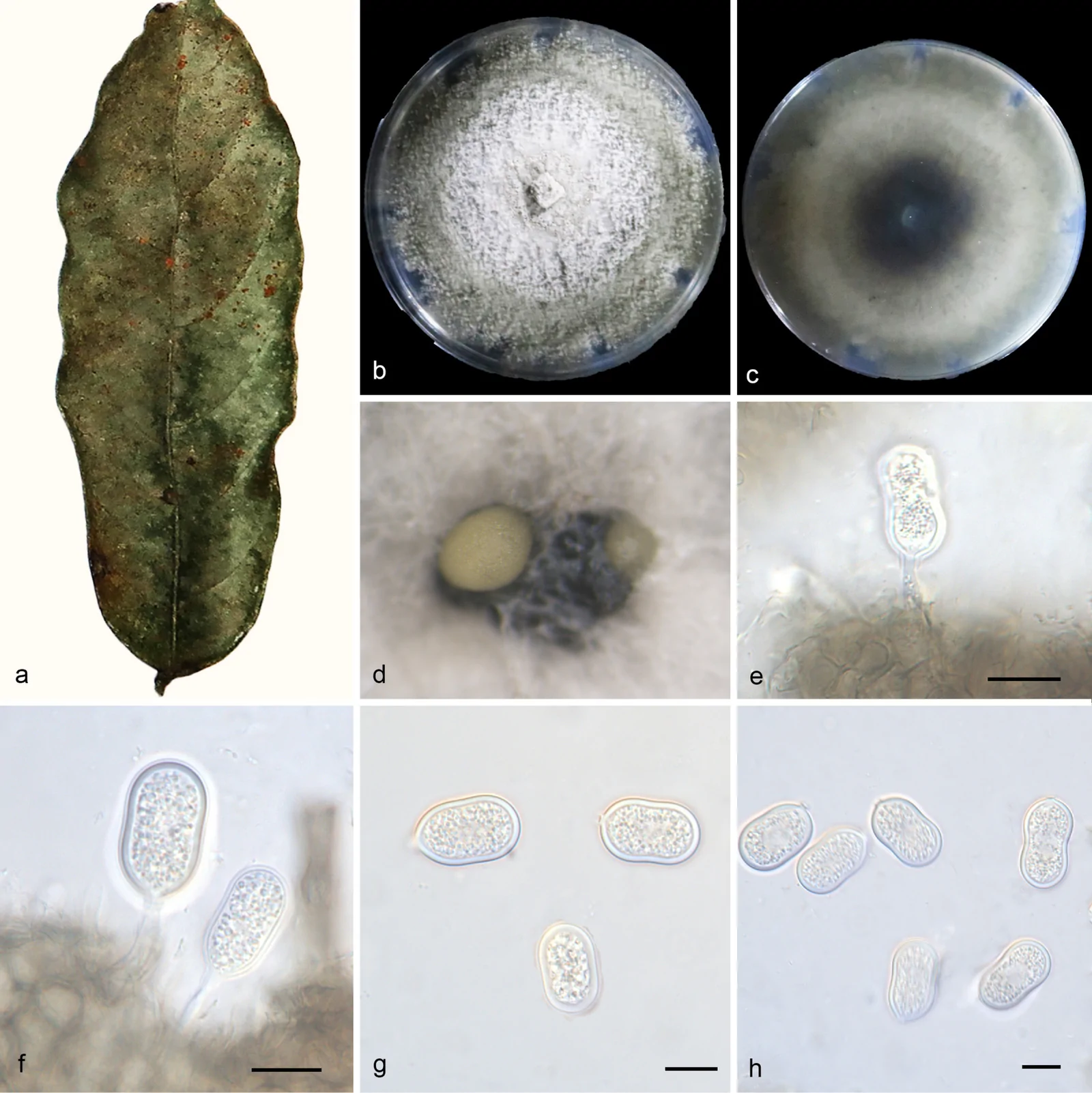
*Pseudoplagiostoma malloti* (SAUCC26144-2-1, ex-type). **a**. A diseased leaf of *Mallotus hookerianus*; **b, c**. Colony from above and below after 14 days on PDA; **d**. Colony overview with conidiomata; **e, f**. Conidiogenous cells with conidia; **g, h**. Conidia. Scale bars: 10 μm (**e–h**).

##### Etymology.

Refers to the host genus *Mallotus*, from which the fungus was isolated.

##### Description.

Asexual morph: Conidiomata pycnidial, solitary, erumpent, globose to pulvinate, dark black at basal wall with pale yellowish-amber ostiolar disc, semi-embedded within dense white cottony aerial mycelium, exuding pale creamy yellow conidial mass at maturity. Conidiophores indistinct, often reduced entirely to conidiogenous cells. Conidiogenous cells hyaline, smooth, lageniform to ampulliform, tapering gradually toward the apex, phialidic, 7.6–15.9 × 2.5–3.2 μm (x̄ = 12.2 × 2.7 μm, *n* = 15). Conidia holoblastic, aseptate, hyaline, smooth, thin-walled, densely granular inside, ellipsoid to broadly oval, straight to slightly obovate, apex obtuse, base tapering to a subtle flat protruding scar, 17.4–22.4 × 9.6–13.8 μm (x̄ = 20.0 × 11.5 μm, *n* = 40). Sexual morph: Not observed.

##### Culture characteristics.

Colonies were cultured on PDA at 25 °C in darkness. After 14 days of cultivation, colonies reached a diameter of 79–87 mm, with daily growth rates of 5.6–6.2 mm. Colonies grew outward in an irregular pattern and were covered with abundant, dense white aerial mycelium over a pale gray substrate. As incubation continued, the colony center gradually deepened to grayish-green, and this pigmentation slowly extended toward the peripheral areas. The reverse side was pale off-white at the initial stage and ultimately formed a distinct dark gray central zone with faint bluish pigment scattered along the margin. Sparse sporulation was observed on PDA after 14 days of incubation.

##### Notes.

Based on phylogenetic analysis combined with five loci (ITS, LSU, *RPB2*, *TEF1-α*, and *TUB2*), *Pseudoplagiostoma malloti* (SAUCC26144-1-1 and SAUCC26144-1-2) forms an independent clade in the phylogenetic trees and is closely related to *Ps. engelhardiae* (CGMCC3.28247 and SAUCC3185B-1). Excluding gap sites, pairwise sequence comparisons between the ex-type strain of *Pseudoplagiostoma malloti* (SAUCC26144-1-1) and the ex-type of *Ps. engelhardiae* (CGMCC3.28247) reveal nucleotide differences of 7.52% (ITS, 44 out of 585 positions), 2.65% (LSU, 22 out of 831 positions), 9.97% (*RPB2*, 89 out of 893 positions), 18.07% (*TEF1-α*, 99 out of 548 positions), and 13.47% (*TUB2*, 61 out of 453 positions). Morphologically, the conidiogenous cells of *Ps. malloti* (7.6–15.9 × 2.5–3.2 μm) are generally wider than those of *Ps. engelhardiae* ((7.5–)11–17.8 × 1.3–2.5 μm). In terms of conidia, *Ps. malloti* produces broader, ellipsoid to broadly oval, densely granular conidia (17.4–22.4 × 9.6–13.8 μm) with subtle flat protruding basal scars, whereas the conidia of *Ps. engelhardiae* are narrower and oblong-fusiform in shape, with sulcate surfaces and distinct basal guttules, and their width (7.8–10.5 μm) is smaller than that of *Ps. malloti*. Therefore, based on morphological distinctions and phylogenetic evidence, this fungus is proposed as *Pseudoplagiostoma malloti* sp. nov., following the species delimitation guidelines of Jeewon and Hyde (2016).

#### Pseudoplagiostoma trachelospermi

Taxon classificationFungiDiaporthalesPseudoplagiostomataceae

J. Y. Sheng, X. G. Zhang & S. Wang sp. nov.

B999AF7D-1C71-5394-A1C2-5A4323B28904

864566

Fig. 5

##### Type.

China • Anhui Province: Shuihou Town, Qianshan City, Anqing City, on diseased leaves of *Trachelospermum jasminoides* Lindl., 27 October 2025, J.Y.Sheng (HMAS 354638, holotype), ex-type living culture SAUCC23445-2-1 = SAUCC23445-2-2.

**Figure 5.**
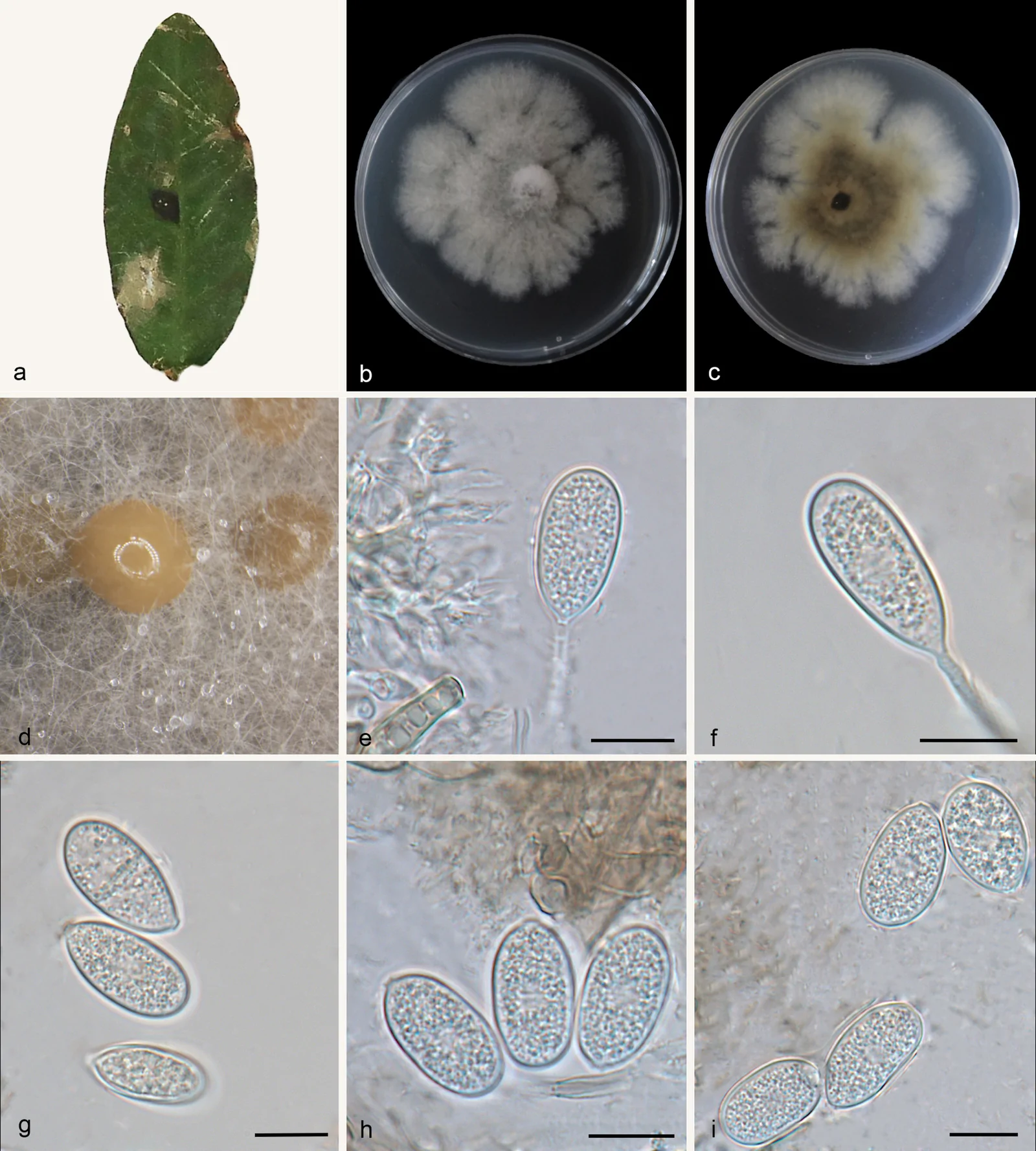
*Pseudoplagiostoma trachelospermi* (SAUCC23445-2-1, ex-type). **a**. A diseased leaf of *Trachelospermum jasminoides*; **b, c**. Colony on PDA from above and below after 7 days; **d**. Colony overview with conidiomata; **e, f**. Conidiogenous cells with conidia; **g, h, i**. Conidia. Scale bars: 10 μm (**e–i**).

##### Etymology.

Refers to the genus *Trachelospermum*, from which the fungus was isolated.

##### Description.

Asexual morph: Conidiomata pycnidial, solitary, erumpent, globose to pulvinate, pale amber to light brown, immersed within dense white cottony aerial mycelium dotted with abundant small transparent vesicles. Conidiophores indistinct, often reduced to conidiogenous cells. Conidiogenous cells hyaline, smooth, lageniform to ampulliform, tapering toward the apex, phialidic, 6.9–14.7 × 01.4–2.6 μm (x̄ = 10.4 × 2.2 μm, *n* = 15). Conidia holoblastic, aseptate, hyaline, smooth, thin-walled, densely granular inside, ellipsoid to broadly oval, straight to slightly obovate, base tapering to a subtle flat scar, 13.6–19.8 × 7.3–11.7 μm (x̄ = 16.9 × 9.0 μm, *n* = 60). Sexual morph: Undetermined.

##### Culture characteristics.

Colonies were cultured on PDA at 25 °C in darkness. After 14 days of cultivation, colonies reached a diameter of 57–69 mm, with daily growth rates of 4.1–4.9 mm. Colonies grew outward in an irregular lobed flower-like pattern and were covered with abundant, fluffy white aerial mycelium. As incubation continued, the colony center gradually turned dark grayish-brown from pure white, and this pigmentation formed a ring-shaped band spreading outward toward the fluffy marginal lobes. The reverse side was uniformly pure white at the initial stage and ultimately developed a prominent dark brown central zone surrounded by a pale off-white periphery. Sparse sporulation was observed on PDA after 21 days of incubation.

##### Notes.

Based on phylogenetic analysis combined with multi-locus sequences (ITS, LSU, *RPB2*, *TEF1-α*, and *TUB2*), *Pseudoplagiostoma trachelospermi* (SAUCC23445-1-1 and SAUCC23445-1-2) forms a distinct and independent clade and is phylogenetically close to three congeners, including *Ps. dipterocarpicola* (MFLUCC 21-0142, MFLUCC 2-0114), *Ps. tonkinense* (CGMCC 3.28243, SAUCC 3186-2), and *Ps. fagacearum* (CFCC 54425, CFCC 54393, CFCC 54410, CFCC 54449, and CFCC 54446). Excluding gap sites from sequence comparisons, the ex-type strain of *Pseudoplagiostoma trachelospermi* (SAUCC23445-1-1) exhibits nucleotide differences from the ex-type strains of these closely related species. Specifically, *Ps. trachelospermi* differs from *Ps. dipterocarpicola* (MFLUCC 21-0142, ex-type) by 5.01% (ITS, 30 out of 599 positions), 1.53% (LSU, 13 out of 849 positions), 16.04% (*TEF1-α*, 47 out of 293 positions), and 12.33% (*TUB2*, 62 out of 503 positions). It differs from *Ps. tonkinense* (CGMCC 3.28243, ex-type) by 4.84% (ITS, 28 out of 579 positions), 1.30% (LSU, 11 out of 845 positions), 12.91% (*RPB2*, 83 out of 643 positions), 19.19% (*TEF1-α*, 99 out of 516 positions), and 12.80% (*TUB2*, 58 out of 453 positions). Additionally, it differs from *Ps. fagacearum* (CFCC 54425, ex-type) by 7.43% (ITS, 42 out of 565 positions), 1.87% (LSU, 15 out of 801 positions), 19.82% (*TEF1-α*, 109 out of 550 positions), and 16.39% (*TUB2*, 80 out of 488 positions). Morphologically, the pycnidia of *Ps. trachelospermi* are pale amber to light brown and embedded within dense white cottony aerial mycelium mixed with abundant transparent vesicles, which is distinct from the black, leathery, epidermal-immersed pycnidia of *Ps. dipterocarpicola*, the slimy black pycnidia of *Ps. tonkinense* growing directly on the agar surface, and the much larger brown pycnidia (400–750 μm diam.) of *Ps. fagacearum*. The conidiogenous cells of *Ps. trachelospermi* measure 6.9–14.7 × 1.4–2.6 μm, wider than the cylindrical conidiogenous cells of *Ps. tonkinense* (4.6–10 × 1.7–3.3 μm) and markedly narrower than those of *Ps. fagacearum* (13.5–18.5 × 4–5.5 μm). In terms of conidia, *Ps. trachelospermi* produces thin-walled, densely granular conidia (13.6–19.8 × 7.3–11.7 μm) with a smooth surface and subtle flat basal scars. In contrast, conidia of *Ps. tonkinense* are narrower and sulcate on the surface, with distinct basal guttules; conidia of *Ps. fagacearum* are thick-walled, multi-guttulate, slightly curved, and constricted at the middle, with a prominent protruding basal hilum and larger overall size. Therefore, based on morphological distinctions and phylogenetic evidence, this fungus is proposed as *Pseudoplagiostoma trachelospermi* sp. nov., following the species delimitation guidelines of Jeewon and Hyde (2016).

### Key to species of *Pseudoplagiostoma*

**Table:** 

1	Sexual morph observed, but asexual morph unknown; ascospores medianly 1-septate, 16–20 × 9–11 µm	***Pseudoplagiostoma machili-nanmu***
–	Asexual morph observed	**2**
2	Conidia 0–2-septate, variable in shape, including reniform, pyriform and obovoid forms	***Ps. jasmini***
–	Conidia aseptate	**3**
3	Conidiomata acervular, subcuticular to subepidermal on leaves, dehiscing by irregular slits	**4**
–	Conidiomata pycnidial	**5**
4	Conidia surrounded by a mucoid sheath	***Ps. corymbiicola***
–	Conidia without a mucoid sheath	***Ps. corymbiae***
5	Conidia exceptionally long, reaching 36 µm	***Ps. dipterocarpi***
–	Conidia not exceeding 27 µm long	**6**
6	Conidia globose, subglobose, or irregularly globose, usually with L/W ratio not exceeding 1.5	**7**
–	Conidia ellipsoid, oblong, elongate ellipsoid, or subcylindrical, usually with L/W ratio exceeding 1.5	**12**
7	Conidia usually less than 14 µm long	**8**
–	Conidia generally 11.5–26 µm long	**10**
8	Conidia 5.3–12.6 × 8.8–11.5 µm, often broader than long	***Ps. jinghongense***
–	Conidia 10.8–13.8 µm long	**9**
9	Conidia hyaline to olivaceous, 11–13.8 × 8–9.4 µm	***Ps. kunmingense***
–	Conidia hyaline, 10.8–13.3 × 8.3–11.1 µm	***Ps. fissistigmatis***
10	Conidia 11.5–18 × 10–12 µm, ellipsoid to globose or irregularly globose; isolated from Fargesia	***Ps. fargesiae***
–	Conidia 17.5–26 µm long	**11**
11	Conidia 17–21 × 13–15 µm, with peg-like hila; sexual morph also known	***Ps. alsophilae***
–	Conidia 17.5–26 × 10–14.5 µm, broad ellipsoid to long-oval or subcylindrical	***Ps. wuyishanense* (syn. *Ps. ilicis* )**
12	Conidia thick-walled, multi-guttulate, often slightly curved and constricted near the middle; conidiomata commonly large	**13**
–	Conidia without this combination of characters	**16**
13	Conidial mass orange; conidia 19–24 × 8.5–10.5 µm	***Ps. neocastanopsidis***
–	Conidial mass brown to dark brown or black	**14**
14	Conidiomata 400–750 µm diam.; conidia 18–22.5 × 10.5–14 µm	***Ps. fagacearum***
–	Conidiomata usually 150–700 µm diam	**15**
15	Conidiomata black, 300–700 µm diam.; conidiogenous cells up to 8.5 µm wide; conidia 16–21.5 × 8.5–11.5 µm	***Ps. quercus***
–	Conidiomata dark brown, 150–400 µm diam.; conidia 17.5–22 × 8.5–13 µm	***Ps. jianfenglingense***
16	Conidiomata 250–380 µm diam.; conidia 17.5–27 × 9–12.5 µm	***Ps. sanmingense***
–	Conidiomata and conidia not as above	**17**
17	Conidia 10–18.5 × 6–9 µm, subcylindrical to elongate ellipsoid	***Ps. fafuense***
–	Conidia otherwise shaped or sized	**18**
18	Conidia frequently constricted at the middle and highly variable in shape, including bean-shaped forms	***Ps. variabile***
–	Conidia not frequently constricted at the middle	**19**
19	Conidia or conidiogenous cells becoming brown at maturity on MEA	***Ps. oldii***
–	Conidia remaining hyaline	**20**
20	Associated with Eucalyptus; conidia 15.5–22 × 6.5–8.2 µm	***Ps. eucalypti***
–	Associated with other hosts	**21**
21	Conidia 14.5–18.8 × 6.1–7.7 µm; conidiogenous cells 4.6–10 × 1.7–3.3 µm	***Ps. tonkinense***
–	Conidia generally broader	**22**
22	Conidia 13–20 × 5.7–7.6 µm, slightly depressed at the middle; from bamboo	***Ps. bambusae***
–	Conidia not depressed at the middle	**23**
23	Conidia 14.7–18 × 6.4–8(–9.5) µm, sulcate; from Altingia	***Ps. altingiae***
–	Conidia not as above	**24**
24	Conidia fusiform to ellipsoid, 16–21.5 × 7.8–10.5 µm, sulcate; from Engelhardia	***Ps. engelhardiae***
–	Conidia broadly oval to ellipsoid, not distinctly fusiform	**25**
25	Conidia 16.5–19.8 × 8.7–12.8 µm; conidiomata black and semi-submerged; from Castanopsis	***Ps. castanopsidis***
–	Conidiomata and conidia not as above	**26**
26	Conidiomata pale brown to dark brown, exuding a pale creamy conidial mass; conidia 14.7–23.1 × 9.4–13.6 µm	***Ps. cinnamomi***
–	Conidial mass and conidia not as above	**27**
27	Conidia 15–23.3 × 9.7–12.7 µm, multi-guttulate; conidiogenous cells slender, 5.4–14.8 × 1.8–3.3 µm	***Ps. indicum***
–	Conidiogenous cells usually wider or conidiomata otherwise distinct	**28**
28	Conidiomata subcuticular to epidermal, 90–150 µm diam.; conidia 16–22 × 9–13 µm	***Ps. mangiferae* (syn. *Ps. diaoluoshanense* )**
–	Conidiomata generally larger or morphology otherwise distinct	**29**
29	Conidia 17.5–23 × 10.5–13.5 µm; from *Machilus*	***Pseudoplagiostoma machili***
–	Conidia narrower or conidiomata distinctive	**30**
30	Conidia 16–20.3 × 7–10.7 µm, oblong to broadly oval; from Ligustrum	***Ps. ligustri***
–	Conidiomata embedded in conspicuous white cottony aerial mycelium, or with a pale yellowish-amber ostiolar disc	**31**
31	Conidiomata pale amber to light brown, embedded in dense white cottony aerial mycelium dotted with transparent vesicles; conidia 13.6–19.8 × 7.3–11.7 µm	***Ps. trachelospermi***
–	Conidiomata dark brown to black at the base, with a pale yellowish-amber ostiolar disc	**32**
32	Conidiomata without dense cottony aerial mycelium; conidia 12.4–20.0 × 7.3–9.8 µm	***Ps. artocarpi***
–	Conidiomata semi-embedded in dense white cottony aerial mycelium and exuding a pale creamy-yellow conidial mass; conidia 17.4–22.4 × 9.6–13.8 µm	***Ps. malloti***

#### Notes

A number of *Pseudoplagiostoma* species could not be included in the key because relevant data are inaccessible due to copyright limitations.

## Discussion

Fungal pathogens causing eucalyptus leaf spot diseases were formerly classified within *Plagiostoma (Gnomoniaceae)* before the genus was formally circumscribed, owing to superficial morphological similarities in their ascomata. Cheewangkoon et al. (2010) separated *Pseudoplagiostoma* from all taxa of *Gnomoniaceae* based on a suite of stable diagnostic morphological traits, including horizontally arranged ascomata with lateral ostiolar necks, asci without amyloid apical rings, septate ascospores with terminal filiform appendages, and distinctive conidiomatal structures. The phylogenetic analyses accompanying the original genus description relied solely on the ITS and LSU loci, which yielded weak resolution for distinguishing cryptic lineages within *Pseudoplagiostoma*. As sequencing technologies advanced, LSU and *TEF1-α* were later incorporated alongside the traditional ITS and *TUB2* markers to build a standard four-locus concatenated matrix (ITS-LSU-*TEF1-α*-*TUB2*). This multi-locus dataset substantially enhanced the phylogenetic resolution for closely related *Pseudoplagiostoma* species and has become the universal framework for taxonomic investigations of this genus (Castlebury et al. 2002; Cheewangkoon et al. 2010; Senanayake et al. 2016, 2017a, 2017b, 2018; Fan et al. 2018; Hyde et al. 2020; Hongsanan et al. 2023).

In the present study, the *RPB2* single-locus phylogeny exhibited low resolution, and closely related *Pseudoplagiostoma* species did not consistently form well-delimited clades with stable support values. *RPB2* was therefore considered unreliable for resolving species-level relationships in the present taxon set and was excluded from the primary species-delimitation framework. In contrast, the ITS, *TEF1-α*, and *TUB2* phylogenetic backbones were highly congruent. The single‑gene trees are shown in Suppl. materials 2–6. The relatively conserved LSU tree showed only minor rearrangements among terminal subclades, representing mild local topological incongruence rather than major lineage shifts. Notably, none of the four newly proposed taxa showed inter-clade movement among the single-locus trees. All were consistently nested within the main *Pseudoplagiostoma* clade and were recovered as distinct lineages in the concatenated phylogeny, with maximal support (MLBS/BYPP = 100/1). These results indicate that the four-locus dataset provides a robust basis for recognizing the novel species.

*Pseudoplagiostoma artocarpi* is closely related to *Ps. quercus*, but it differs consistently in conidial morphology. Conidia of *Ps. quercus* are thick-walled, multi-guttulate, ellipsoid to oblong-cylindrical, slightly constricted at the middle, and gently curved, with a prominent basal hilum. In contrast, conidia of *Ps. artocarpi* are thin-walled, densely granular internally, ellipsoid to broadly oval, and lack both median constriction and curvature. They also possess only a subtle flat basal scar. Differences in conidial dimensions and length/width ratios further separate these taxa. The molecular and morphological evidence therefore supports *Ps. artocarpi* as a distinct species. *Pseudoplagiostoma cinnamomi* was recovered as the sister species of *Ps. alsophilae*. However, the two taxa are consistently distinguishable morphologically. Only the asexual morph was observed in *Ps. cinnamomi*, whereas *Ps. alsophilae* possesses a mature sexual morph. In addition, *Ps. alsophilae* produces globose conidia with peg-like basal hila and guttulate contents, while *Ps. cinnamomi* has ellipsoid to broadly oval conidia lacking peg-like hila, instead bearing only a subtle, flat basal scar. The conidia of *Ps. cinnamomi* also contain densely granular contents and have a smaller mean width. These stable morphological differences, together with their molecular divergence, support the recognition of *Ps. cinnamomi* as an independent species. *Pseudoplagiostoma malloti* is phylogenetically sister to *Ps. engelhardiae*, but the two species differ in both cultural and conidial characters. *Pseudoplagiostoma engelhardiae* forms concentric, wheel-shaped colonies on PDA and produces oblong-fusiform, sulcate conidia with distinct basal guttules. In contrast, *Ps. malloti* produces ellipsoid to broadly oval conidia that lack surface sulci, contain densely granular contents, and possess only a subtle, flat, protruding basal scar. These diagnostic conidial and cultural differences, combined with the well-supported phylogenetic separation, confirm *Ps. malloti* as a distinct species. *Pseudoplagiostoma trachelospermi* is phylogenetically related to the clade comprising *Ps. tonkinense* and *Ps. dipterocarpicola*, but it can be distinguished from both species by stable morphological characters. Conidia of *Ps. tonkinense* are sulcate, whereas *Ps. dipterocarpicola* has distinctly longer conidiogenous cells and thick-walled conidia with a broad length range. In contrast, *Ps. trachelospermi* produces smooth, thin-walled, and densely granular conidia without surface sulci, has moderately sized conidiogenous cells, and shows a narrower conidial length range. In culture, *Ps. tonkinense* develops pale-gray, fish-scale-like colonies on PDA, a feature not observed in the other two species. These differences, together with their molecular divergence, support the recognition of *Ps. trachelospermi* as a separate species.

Single-locus phylogenies (ITS, LSU, *TEF1-α*, and *TUB2*) and the combined multi-locus tree consistently separate the newly sequenced isolates from members of *Pyrispora*. Although the present taxon sampling was designed primarily for species delimitation within *Pseudoplagiostoma*, and *Pyrispora* was used as the outgroup, the analyses clearly demonstrate that the four novel taxa do not belong to *Pyrispora*. Broader diaporthalean studies have likewise supported *Pseudoplagiostoma* and *Pyrispora* as phylogenetically close but distinct lineages assigned to *Pseudoplagiostomataceae* and *Pyrisporaceae*, respectively (Jiang et al. 2025). The morphological distinction between the two genera is consistent with their molecular separation. Species of *Pseudoplagiostoma* generally possess ascospores that are 1-septate at the median and have terminal appendages in the sexual morph and typically produce thick-walled conidia with conspicuous hila in the asexual morph. In contrast, *Pyrispora* is characterized by aseptate ascospores and lacks the thick-walled conidia typical of *Pseudoplagiostoma*.

Recent studies have recognized *Pseudoplagiostoma* as an ecologically diverse group of fungi associated with woody plants. Species have been reported as leaf-spot-associated fungi, endophytes, and saprobes on a broad range of hosts (Suwannarach et al. 2016; Wang et al. 2016; Silva et al. 2023; Zhang et al. 2023; Haituk et al. 2024; Wu et al. 2024). In the present study, four novel taxa were isolated from symptomatic leaves of *Artocarpus hainanensis* (*Moraceae*), *Cinnamomum* sp. (*Lauraceae*), *Mallotus hookerianus* (*Euphorbiaceae*), and *Trachelospermum jasminoides* (*Apocynaceae*). The records from *Moraceae*, *Euphorbiaceae*, and *Apocynaceae* expand the known host-family range of *Pseudoplagiostoma*. Each novel species was recovered from a single host species, and the present data therefore demonstrate host breadth at the generic level rather than strict host specificity at the species level. The occurrence of different *Pseudoplagiostoma* lineages on phylogenetically distant woody hosts suggests that the genus has considerable ecological flexibility. However, cross-inoculation experiments and sampling from alternative hosts are required to determine whether each species is host-specific, host-preferential, or capable of colonizing a wider range of plants (Hyde et al. 2024a, 2024b).

The four novel taxa were collected from symptomatic leaves in subtropical China, indicating their occurrence in warm and humid leaf-associated habitats. However, field occurrence cannot by itself establish thermal optima or tolerance limits. Prior studies on *Ps. eucalypti* have indicated that high humidity promotes disease progression. Its vegetative growth and sporulation peak at approximately 25–26 °C, while temperatures ≥32 °C tend to suppress disease development (Sankaran et al. 1995; Cheewangkoon et al. 2010). Such information should not be extrapolated directly to the four novel species because temperature-dependent growth and sporulation were not assessed in the present study. Their thermal adaptability should therefore be interpreted only as an inferred association with subtropical environmental conditions.

Furthermore, because artificial inoculation assays were not performed, the present isolates should be regarded as fungi associated with leaf-spot lesions rather than confirmed primary pathogens. They may function as pathogens, opportunistic colonizers, or latent endophytes, depending on host condition and local environmental factors. Future studies should include pathogenicity assays, cross-inoculation tests, radial-growth and sporulation experiments across a defined temperature gradient, and multi-season field surveys that record temperature, rainfall, relative humidity, and leaf-wetness duration. Such studies will clarify the host range, pathogenicity, thermal response, and ecological roles of these newly described *Pseudoplagiostoma* species.

## Supplementary Material

XML Treatment for Pseudoplagiostoma artocarpi

XML Treatment for Pseudoplagiostoma cinnamomi

XML Treatment for Pseudoplagiostoma malloti

XML Treatment for Pseudoplagiostoma trachelospermi

## Additional information

### Artificial Intelligence (AI) use

The authors accept full responsibility for the content of the manuscript, including the disclosure of any use of AI.

No AI tools were used in the preparation of this manuscript.

### Author ORCIDs

J.-Y. Sheng https://orcid.org/0009-0003-3882-6748

Y. Jiang https://orcid.org/0009-0003-1292-610X

X.-G. Yan https://orcid.org/0009-0005-6257-3490

X.-S. Wang https://orcid.org/0009-0005-1127-7368

Y. Zhang https://orcid.org/0009-0002-9239-8097

C.-C. Ai https://orcid.org/0009-0002-3934-7944

X.-G. Zhang https://orcid.org/0000-0001-9733-8494

S. Wang https://orcid.org/0000-0002-7376-7638

### Data availability

All of the data that support the findings of this study are available in the main text or Supplementary Information.

## Supplementary materials

Supplementary material 1 GenBank accession numbers of the taxa in the phylogenetic reconstruction Data type docx https://binary.pensoft.net/file/1758257This dataset is made available under the Open Database License (http://opendatacommons.org/licenses/odbl/1.0/). The Open Database License (ODbL) is a license agreement intended to allow users to freely share, modify, and use this Dataset while maintaining this same freedom for others, provided that the original source and author(s) are credited. Jin-Yan Sheng, Yang Jiang, Xi-Gang Yan, Xing-Sheng Wang, Yue Zhang, Cong-Cong Ai, Xiu-Guo Zhang, Shi Wang

Supplementary material 2 ITS single-gene phylogenetic tree Data type jpg https://binary.pensoft.net/file/1758258This dataset is made available under the Open Database License (http://opendatacommons.org/licenses/odbl/1.0/). The Open Database License (ODbL) is a license agreement intended to allow users to freely share, modify, and use this Dataset while maintaining this same freedom for others, provided that the original source and author(s) are credited. Jin-Yan Sheng, Yang Jiang, Xi-Gang Yan, Xing-Sheng Wang, Yue Zhang, Cong-Cong Ai, Xiu-Guo Zhang, Shi Wang

Supplementary material 3 LSU single-gene phylogenetic tree Data type jpg https://binary.pensoft.net/file/1758259This dataset is made available under the Open Database License (http://opendatacommons.org/licenses/odbl/1.0/). The Open Database License (ODbL) is a license agreement intended to allow users to freely share, modify, and use this Dataset while maintaining this same freedom for others, provided that the original source and author(s) are credited. Jin-Yan Sheng, Yang Jiang, Xi-Gang Yan, Xing-Sheng Wang, Yue Zhang, Cong-Cong Ai, Xiu-Guo Zhang, Shi Wang

Supplementary material 4 *TEF1-α* single-gene phylogenetic tree Data type jpg https://binary.pensoft.net/file/1758260This dataset is made available under the Open Database License (http://opendatacommons.org/licenses/odbl/1.0/). The Open Database License (ODbL) is a license agreement intended to allow users to freely share, modify, and use this Dataset while maintaining this same freedom for others, provided that the original source and author(s) are credited. Jin-Yan Sheng, Yang Jiang, Xi-Gang Yan, Xing-Sheng Wang, Yue Zhang, Cong-Cong Ai, Xiu-Guo Zhang, Shi Wang

Supplementary material 5 TUB single-gene phylogenetic tree Data type jpg https://binary.pensoft.net/file/1758261This dataset is made available under the Open Database License (http://opendatacommons.org/licenses/odbl/1.0/). The Open Database License (ODbL) is a license agreement intended to allow users to freely share, modify, and use this Dataset while maintaining this same freedom for others, provided that the original source and author(s) are credited. Jin-Yan Sheng, Yang Jiang, Xi-Gang Yan, Xing-Sheng Wang, Yue Zhang, Cong-Cong Ai, Xiu-Guo Zhang, Shi Wang

Supplementary material 6 *RPB2* single-gene phylogenetic tree Data type jpg https://binary.pensoft.net/file/1758262This dataset is made available under the Open Database License (http://opendatacommons.org/licenses/odbl/1.0/). The Open Database License (ODbL) is a license agreement intended to allow users to freely share, modify, and use this Dataset while maintaining this same freedom for others, provided that the original source and author(s) are credited. Jin-Yan Sheng, Yang Jiang, Xi-Gang Yan, Xing-Sheng Wang, Yue Zhang, Cong-Cong Ai, Xiu-Guo Zhang, Shi Wang
