# Supplementary material for: Four new species of Pseudoplagiostoma (Pseudoplagiostomataceae, Diaporthales) based on morphology and multi-locus phylogeny from China
Source: MycoKeys. 2026 Aug 27;139:251–76. doi: 10.3897/mycokeys.139.207860 (PMC13542766; doi:10.3897/mycokeys.139.207860)
Supplement: Supplementary file 1 [file mycokeys-139-251-s001.docx]

**Supplementary Table S1.** GenBank accession numbers of the taxa in the phylogenetic reconstruction.

| Species | Strains | GenBank Accession Numbers | | | | | References |
| --- | --- | --- | --- | --- | --- | --- | --- |
|  |  | ITS | LSU | *TEF1-α* | *TUB2* | *RPB2* |  |
| *Pseudoplagiostoma alsophilae* | SAUCC WZ0451* | OP810625 | OP810631 | OP828580 | OP828586 | OP828578 | Zhang et al.2023 |
| *Ps. alsophilae* | SAUCC WZ0152 | OP810626 | OP810632 | OP828581 | OP828587 | OP828579 | Zhang et al.2023 |
| *Ps. altingiae* | CGMCC 3.28244* | PQ338993 | PQ339638 | PQ358326 | PQ358352 | PQ367345 | Zhang et al.2025 |
| *Ps. altingiae* | CGMCC 3.28245 | PQ338992 | PQ339637 | PQ358325 | PQ358351 | PQ367344 | Zhang et al.2025 |
| *Ps. altingiae* | CGMCC 3.28246 | PQ338991 | PQ339636 | PQ358324 | PQ358350 | PQ367343 | Zhang et al.2025 |
| *Ps. altingiae* | CGMCC 3.28242 | PP101224 | PP355149 | PP379646 | PP379689 | PP379569 | Zhang et al.2025 |
| ***Ps. artocarpi*** | **SAUCC26109-1-1*** | **PZ520514** | **PZ520523** | **PZ577218** | **PZ570107** | **PZ622795** | **This study** |
| ***Ps. artocarpi*** | **SAUCC26109-1-2** | **PZ520515** | **PZ520524** | **PZ577219** | **PZ570108** | **PZ622796** | **This study** |
| *Ps. bambusae* | CGMCC 3.25363 | OR623171 | OR623238 | OR637439 | OR637423 | OR637431 | Zhang et al.2023 |
| *Ps. bambusae* | CGMCC 3.25364 | OR623172 | OR623239 | OR637440 | OR637424 | OR637432 | Zhang et al.2023 |
| *Ps. bambusae* | SAUCC 1206-4* | OP810629 | OP810635 | OP828584 | OP828590 | NA | Zhang et al.2023 |
| *Ps. bambusae* | SAUCC 1206-6 | OP810630 | OP810636 | OP828585 | OP828591 | NA | Zhang et al.2023 |
| *Ps. castanopsidis* | CGMCC 3.28235* | PP446505 | PP455436 | PP453694 | PP453702 | PP453711 | Zhang et al.2025 |
| *Ps. castanopsidis* | SAUCC 3969-1 | PP101225 | PP355150 | PP379647 | PP379690 | PP379570 | Zhang et al.2025 |
| ***Ps. cinnamomi*** | **SAUCC22696-1-1*** | **PZ520510** | **PZ520519** | **PZ562017** | **PZ573184** | **PZ573188** | **This study** |
| ***Ps. cinnamomi*** | **SAUCC22696-1-2** | **PZ520511** | **PZ520520** | **PZ562018** | **PZ573185** | **PZ573189** | **This study** |
| *Ps. corymbiae* | CBS 132529* | JX069861 | JX069845 | NA | NA | NA | Crous et al.2012c |
| *Ps. corymbiicola* | CBS 145052* | MK047425 | MK047476 | MK047558 | MK047577 | NA | Crous et al.2018 |
| *Ps. dipterocarpi* | TBRC 1895* | KR994682 | KR994683 | NA | NA | NA | Suwannarach et al. 2016 |
| *Ps. dipterocarpicola* | MFLUCC 21-0114 | OM228843 | OM228841 | OM219628 | OM219637 | NA | Tang et al.2022 |
| *Ps. dipterocarpicola* | MFLUCC 21-0142* | OM228844 | OM228842 | OM219629 | OM219638 | NA | Tang et al.2022 |
| *Ps. engelhardiae* | CGMCC 3.28247* | PQ338989 | PQ339634 | PQ358322 | PQ358348 | PQ367341 | Zhang et al.2025 |
| *Ps. engelhardiae* | SAUCC 3185B–1 | PQ338990 | PQ339635 | PQ358323 | PQ358349 | PQ367342 | Zhang et al.2025 |
| *Ps. eucalypti* | CPC 14161 | GU973510 | GU973604 | GU973540 | GU973573 | NA | Cheewangkoonet al. 2010 |
| *Ps. eucalypti* | CBS 124807* | GU973512 | GU973606 | GU973542 | GU973575 | NA | Cheewangkoon et al. 2010 |
| *Ps. eucalypti* | SAUCC 4780 | PP101200 | PP355146 | PP379643 | PP379686 | PP379566 | Zhang et al.2025 |
| *Ps. fafuense* | CGMCC 3.25357* | OR623165 | OR623232 | OR637433 | OR637417 | OR637425 | Mu et al. 2024b |
| *Ps. fafuense* | CGMCC 3.25358 | OR623166 | OR623233 | OR637434 | OR637418 | OR637426 | Mu et al. 2024b |
| *Ps. fagacearum* | CFCC 54425* | OK339766 | OK339795 | OK358605 | OK358617 | NA | Jiang et al. 2025 |
| *Ps. fagacearum* | CFCC 54446 | OK339767 | OK339796 | OK358606 | OK358618 | NA | Jiang et al. 2025 |
| *Ps. fagacearum* | CFCC 54410 | OK339768 | OK339797 | OK358607 | OK358619 | NA | Jiang et al. 2025 |
| *Ps. fagacearum* | CFCC 54449 | OK339769 | OK339798 | OK358608 | OK358620 | NA | Jiang et al. 2025 |
| *Ps. fagacearum* | CFCC 54393 | OK339770 | OK339799 | OK358609 | OK358621 | NA | Jiang et al. 2025 |
| *Ps. fargesiae* | CGMCC 3.28252* | PQ338987 | PQ339632 | PQ358320 | PQ358346 | PQ367339 | Zhang et al.2025 |
| *Ps. fargesiae* | SAUCC 5577-1 | PQ338988 | PQ339633 | PQ358321 | PQ358347 | PQ367340 | Zhang et al.2025 |
| *Ps. fssistigmatis* | CGMCC 3.28261* | PP446502 | PP355144 | PP379641 | PP379684 | PP379565 | Zhang et al.2025 |
| *Ps. fssistigmatis* | SAUCC 5568-1 | PP446501 | PP455433 | PP453699 | PP453700 | PP453709 | Zhang et al.2025 |
| *Ps. ilicis* | CGMCC 3.25359 | OR623167 | OR623234 | OR637435 | OR637419 | OR637427 | Mu et al. 2024b |
| *Ps. ilicis* | CGMCC 3.25360* | OR623168 | OR623235 | OR637436 | OR637420 | OR637428 | Mu et al. 2024b |
| *Ps. indicum* | CGMCC 3.28238* | PP101202 | PP355156 | PP379652 | PP379696 | PQ367346 | Zhang et al.2025 |
| *Ps. indicum* | SAUCC 3269 | PP446500 | PP455438 | PP453695 | PP453704 | PQ367347 | Zhang et al.2025 |
| *Ps. inthanonense* | MFLU 23-0345* | NR_191298 | NG_243399 | OR650831 | OR611920 | OR611921 | Silva et al.2023 |
| *Ps. jasmini* | MFLUCC 23-0044* | OQ786078 | OQ786079 | OQ850145 | OQ850148 | NA | Gomdola et al.2023 |
| *Ps. jianfenglingense* | CGMCC 3.28254* | PQ338983 | PQ339628 | PQ358316 | PQ358342 | PQ367335 | Zhang et al.2025 |
| *Ps. jianfenglingense* | SAUCC 3971 | PQ338984 | PQ339629 | PQ358317 | PQ358343 | PQ367336 | Zhang et al.2025 |
| *Ps. jianfenglingense* | CFCC 54396 | OK339764 | OK339793 | OK358603 | OK358615 | NA | Jiang et al. 2025 |
| *Ps. jianfenglingense* | CFCC 55894 | OK339765 | OK339794 | OK358604 | OK358616 | NA | Jiang et al. 2025 |
| *Ps. jinghongense* | CGMCC 3.28265* | PP101237 | PP355169 | PP379665 | PP379709 | PP379586 | Zhang et al.2025 |
| *Ps. jinghongense* | SAUCC 2287-5 | PP446509 | PP455441 | PP453698 | PP453707 | PP453715 | Zhang et al.2025 |
| *Ps. kunmingense* | CGMCC 3.28240* | PP101222 | PP355145 | PP379642 | PP379685 | PP379588 | Zhang et al.2025 |
| *Ps. kunmingense* | SAUCC 5402-2 | PP446503 | PP455434 | PP453692 | PP453708 | PP453716 | Zhang et al.2025 |
| *Ps. ligustri* | CGMCC 3.28241* | PP101226 | PP355152 | PQ358327 | PP379692 | PP379572 | Zhang et al.2025 |
| *Ps. ligustri* | SAUCC 3790-1 | PP446506 | PP455437 | PQ358328 | PP453703 | PP453712 | Zhang et al.2025 |
| *Ps. machili* | SAUCC BW0233* | OP810627 | OP810633 | OP828582 | OP828588 | NA | Zhang et al.2025 |
| *Ps. machili* | SAUCC BW0221 | OP810628 | OP810634 | OP828583 | OP828589 | NA | Zhang et al.2025 |
| *Ps. machili-nanmu* | CGMCC 3.28253* | PQ338985 | PQ339630 | PQ358318 | PQ358344 | PQ367337 | Zhang et al.2025 |
| *Ps. machili-nanmu* | SAUCC 1611 | PQ338986 | PQ339631 | PQ358319 | PQ358345 | PQ367338 | Zhang et al.2025 |
| ***Ps. malloti*** | **SAUCC26144-2-1*** | **PZ520516** | **PZ520525** | **PZ577220** | **PZ573186** | **PZ631299** | **This study** |
| ***Ps. malloti*** | **SAUCC26144-2-2** | **PZ520517** | **PZ520526** | **PZ577221** | **PZ573187** | **PZ631300** | **This study** |
| *Ps. mangiferae* | KUMCC 18-0179* | MK084824 | MK084825 | NA | NA | NA | Phookamsak etal. 2019 |
| *Ps. mangiferae (syn. Ps. diaoluoshanense)* | CGMCC 3.28236* | PP101232 | PP355161 | PP379657 | PP379701 | PP379578 | Zhang et al.2025 |
| *Ps. mangiferae (syn. Ps. diaoluoshanense)* | SAUCC 2892 | PP446508 | PP455440 | PP453697 | PP453706 | PP453714 | Zhang et al.2025 |
| *Ps. neocastanopsidis* | CFCC 54447* | OK339762 | OK339791 | OK358601 | OK358613 | NA | Jiang et al. 2025 |
| *Ps. neocastanopsidis* | CFCC 52809 | OK339763 | OK339792 | OK358602 | OK358614 | NA | Jiang et al. 2025 |
| *Ps. oldii* | CBS 124808* | GU973534 | GU973609 | GU973564 | GU993862 | NA | Cheewangkoonet al. 2010 |
| *Ps. oldii* | CBS 115722 | GU973535 | GU973610 | GU973565 | GU993864 | NA | Cheewangkoon et al. 2010 |
| *Ps. perseae* | BCRC FU31388* | MT233359 | MT233373 | NA | MT251143 | NA | Wu et al. 2024 |
| *Ps. perseae* | BCRC FU31389 | MT233360 | MT233374 | NA | MT251144 | NA | Wu et al. 2024 |
| *Ps. quercus* | CFCC 55232* | OK339771 | OK339800 | OK358610 | OK358622 | NA | Jiang et al. 2025 |
| *Ps. quercus* | CFCC 55192 | OK339772 | OK339801 | OK358611 | OK358623 | NA | Jiang et al. 2025 |
| *Ps. quercus* | CFCC 55262 | OK339773 | OK339802 | OK358612 | OK358624 | NA | Jiang et al. 2025 |
| *Ps. sanmingense* | CGMCC 3.25361 | OR623169 | OR623236 | OR637437 | OR637421 | OR637429 | Mu et al. 2024b |
| *Ps. sanmingense* | CGMCC 3.25362* | OR623170 | OR623237 | OR637438 | OR637422 | OR637430 | Mu et al. 2024b |
| *Ps. tonkinense* | CGMCC 3.28243* | PP101229 | PP355158 | PP379654 | PP379698 | PP379575 | Zhang et al.2025 |
| *Ps. tonkinense* | SAUCC 3186-2 | PP446507 | PP455439 | PP453696 | PP453705 | PP453713 | Zhang et al.2025 |
| ***Ps.trachelospermi*** | **SAUCC23445-2-1*** | **PZ520512** | **PZ520521** | **PZ577216** | **PZ570107** | **PZ622793** | **This study** |
| ***Ps.trachelospermi*** | **SAUCC23445-2-2** | **PZ520513** | **PZ520522** | **PZ577217** | **PZ570108** | **PZ622794** | **This study** |
| *Ps. variabile* | CBS 113067* | GU973536 | GU973611 | GU973566 | GU993863 | NA | Cheewangkoon et al. 2010 |
| *Ps. wuyishanense* | CGMCC 3.25367 | PP658312 | NA | NA | PP665723 | PP665719 | Mu et al. 2024a |
| *Ps. wuyishanense* | CGMCC 3.25368 * | PP658313 | NA | NA | PP665724 | PP665720 | Mu et al. 2024a |
| *Ps. wuyishanense (syn. Ps. ilicis)* | CGMCC 3.25359 | OR623167 | OR623234 | OR637435 | OR637419 | OR637427 | Mu et al. 2024b |
| *Ps. wuyishanense (syn. Ps. ilicis)* | CGMCC 3.25360 | OR623168 | OR623235 | OR637436 | OR637420 | OR637428 | Mu et al. 2024b |
| *castaneae* | CFCC 54349 * | MW208108 | MW208105 | MW227340 | NA | MW218535 | Jiang et al.2021a |
| *Pyrispora castaneae* | CFCC 54350 | MW208109 | MW208106 | MW227341 | NA | MW218536 | Jiang et al.2021a |
| *Py. castaneae* | SAUCC my0162 | MZ156982 | MZ156985 | MZ220321 | MZ220325 | MZ220323 | Mu et al. 2022 |
| *Py. castaneae* | SAUCC my0523 | MZ156983 | MZ156986 | MZ220322 | MZ220326 | MZ220324 | Mu et al. 2022 |
| *Py. humilis (syn. Ps. humilis)* | CCUB 5837* | OR452735 | NA | OR540677 | OR420725 | NA | Magalhães et al. 2024 |
| *Py. humilis (syn. Ps. humilis)* | CCUB 5838 | OR452736 | NA | OR540678 | OR420726 | NA | Magalhães et al. 2024 |
| *Py. myracrodruonis (syn. Ps. myracrodruonis)* | URM 7799* | MG870421 | MK982151 | MK982557 | MN019566 | MK977723 | Bezerra et al.2019 |
| *Py. myracrodruonis (syn. Ps. myracrodruonis)* | URM 8123 | MK982150 | MK982152 | MK982558 | MN019567 | MK977724 | Bezerra et al.2019 |
| *Py. quercicola* | CFCC 54868* | OK339774 | OK339803 | OK358589 | NA | OK358591 | Jiang et al. 2025 |
| *Py. quercicola* | CFCC 54375 | OK339775 | OK339804 | OK358590 | NA | OK358592 | Jiang et al. 2025 |

**Notes**: New species established in this study are shown in bold. Those marked “*” in the table are represented as ex-type or ex-epitype strains. “NA”: Not available.
